# Recurrent Left Periprosthetic Posterior Knee Dislocation in an Elderly Woman With Dementia and Altered Mental Status: A Case Report

**DOI:** 10.7759/cureus.66031

**Published:** 2024-08-02

**Authors:** Blake E Delgadillo, Zachary J Buchman, Aaron Brown, Justin R Federico

**Affiliations:** 1 Orthopaedic Surgery, Lake Erie College of Osteopathic Medicine, Bradenton, USA; 2 Internal Medicine, Baptist Health, Jacksonville, USA

**Keywords:** operative planning, elderly patients, rehabilitation, revision joint replacement, orthopedic injury, rotating hinged knee prosthesis, altered mental state, knee dislocation, total knee revision arthroplasty, total knee arthroplasty

## Abstract

The case presented in this article is one of recurrent left posterior periprosthetic knee dislocation (PPKD) in a patient with altered mental status (AMS). The patient, a 69-year-old female with a complex medical history including dementia, Ménière’s syndrome, and left total knee arthroplasty, presented to the emergency department with AMS whereupon a left PPKD was discovered. Less than three weeks before this presentation, she sustained a left PPKD during a previous admission. During her current admission, she sustained yet another left PPKD after trials of closed reduction and immobilization. The patient eventually underwent a left cemented revision total knee arthroplasty with a hinged prosthesis. The implant was noted to be stable, and the patient had minimal pain postoperatively with no vascular or neurological injury. Upon outpatient follow-up, the patient reported doing well. There have been few documented cases of recurrent or chronic PPKD in individuals with AMS or restricted intellect. These comorbidities create a complex approach to diagnosing and treating the aforementioned orthopedic injury, and as this injury can have devastating consequences, quickly and effectively delivering diagnosis and treatment is vital. This case highlights the importance of early identification, risk factors, preoperative management, and appropriate operative course for patients with AMS and recurrent PPKDs.

## Introduction

In the United States, total knee arthroplasty (TKA) procedures are relatively common with 234.2 completed per 100,000 population (female-to-male ratio of 3:2) in 2008 [1]. At that time, the rate of TKA had grown by 6.8% over the 10 years prior. Of these, roughly 10% were considered revision surgeries meant to address unsatisfactory results or complications [1]. In 2014, the most common causes necessitating revision TKA included prosthetic joint infection (42.7%), aseptic loosening (32.2%), and joint instability (13.1%) [2]. One manifestation of joint instability presents as posterior joint dislocation, which is the subluxation of the proximal tibia posterior to the distal femur. Risk factors for such a dislocation include obesity, neuropsychiatric disorders, peripheral neuromuscular diseases, and ligamentous lesions [3]. These dislocations pose a considerable threat as complications can include common peroneal nerve injury (25%), vascular insult (18%), and even amputation (12%) in standard patients. In those with prior TKA, complications include chronic periprosthetic infection, implant loosening, bone loss, and flexion contracture [3-5]. However, after TKA using newer designs, periprosthetic knee dislocations are rare (0.15-0.5%), with obesity as the most common comorbidity [3,6]. These risks are heightened in those with cognitive impairment, who may have delayed or undiagnosed dislocations. In such cases, to reduce the risk of further complications, revision surgery utilizing a hinged prosthesis can be used. This style of prosthesis is the most constrained type of knee replacement prosthesis and is generally associated with poorer outcomes and higher rates of complications in the general population, leaving their use for select cases [7]. Due to the low rate of use of hinged prostheses and the rarity of chronic posterior TKA dislocation requiring revision surgery, there is little in terms of documented cases of such, especially in individuals with cognitive impairment [4,8]. Therefore, our objective is to provide a reference point for such an occurrence that may help guide surgeons with similar cases in the future. The case presented here is of an elderly woman with dementia, altered mental status (AMS), TKA, and multiple recent posterior periprosthetic knee dislocations (PPKDs) that resulted in revision TKA with a hinged prosthesis, which highlights the complexity of managing recurrent dislocations in vulnerable populations.

## Case presentation

**Figure 1:**
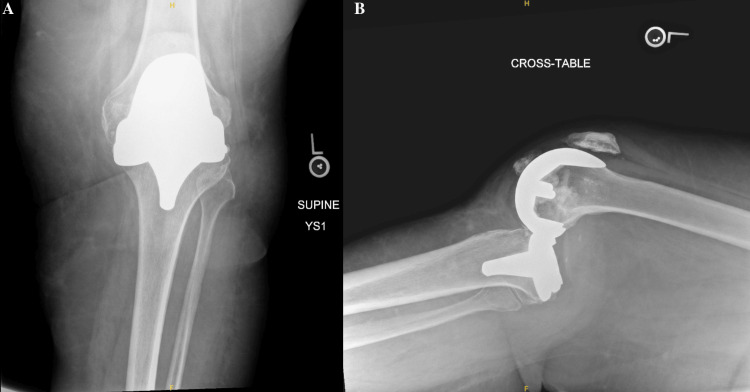
Anteroposterior and cross-table radiographs of the left knee with periprosthetic knee dislocation. (A) Anteroposterior radiograph of the left knee showing moderate soft tissue edema. No acute fracture is evident. (B) Cross-table radiograph of the left knee showing a posterior periprosthetic knee dislocation with moderate soft tissue edema and heterotopic ossification adjacent to the patellar tendon. No evidence of fracture can be seen.

**Figure 2:**
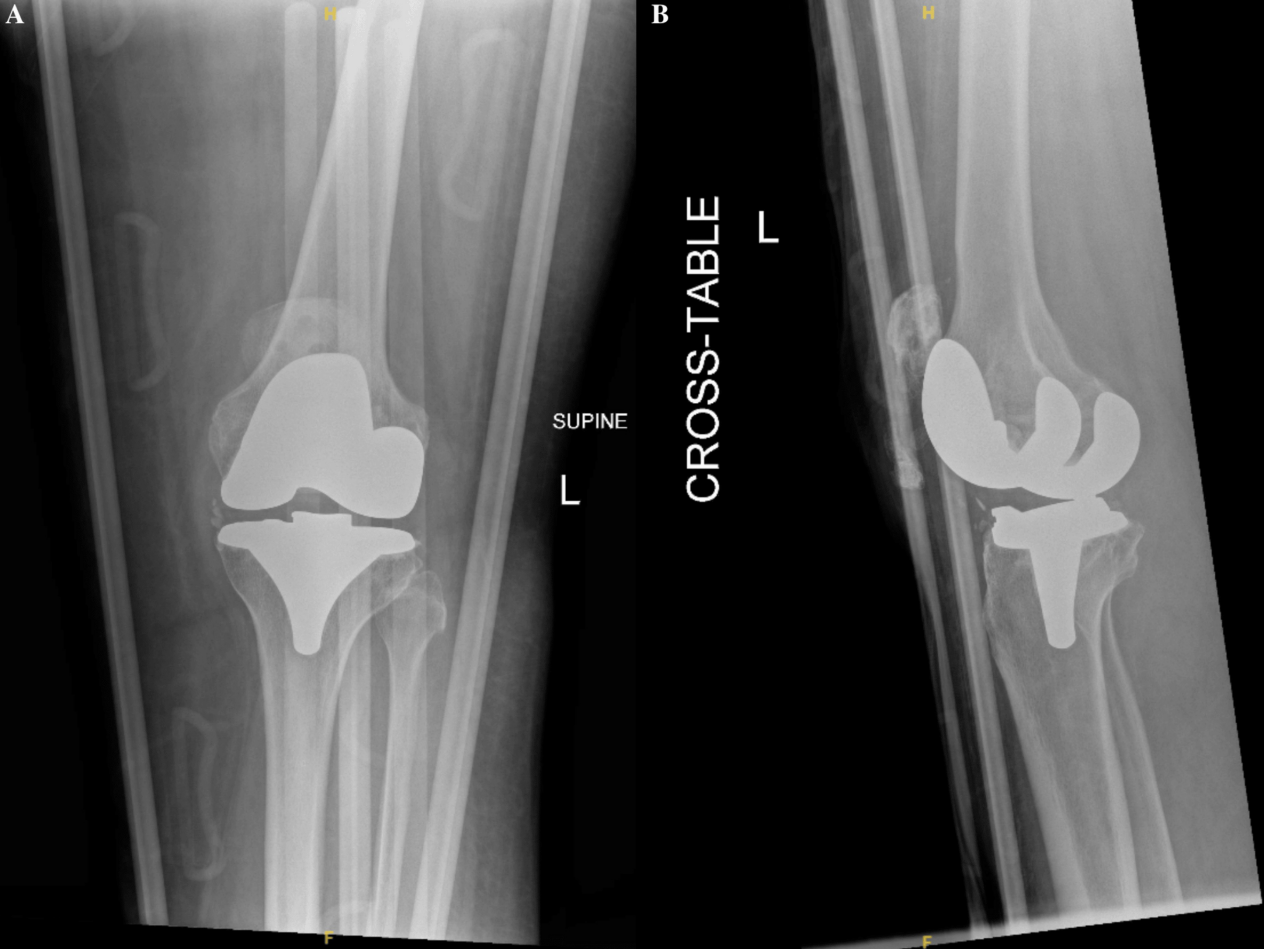
Anteroposterior and cross-table radiographs of the left knee status post-closed reduction. (A) Anteroposterior radiograph of the left knee showing satisfactory reduction and alignment status post-closed reduction in the emergency department. There is evidence of demineralized bone, but no fracture is noted. An external knee immobilizer is seen. (B) Cross-table radiograph of the left knee showing satisfactory reduction and alignment status post-closed reduction in the emergency department. There is no evidence of fracture, but demineralized bone is noted. Heterotopic ossification of the patellar tendon is appreciated. An external knee immobilizer is seen.

**Figure 3:**
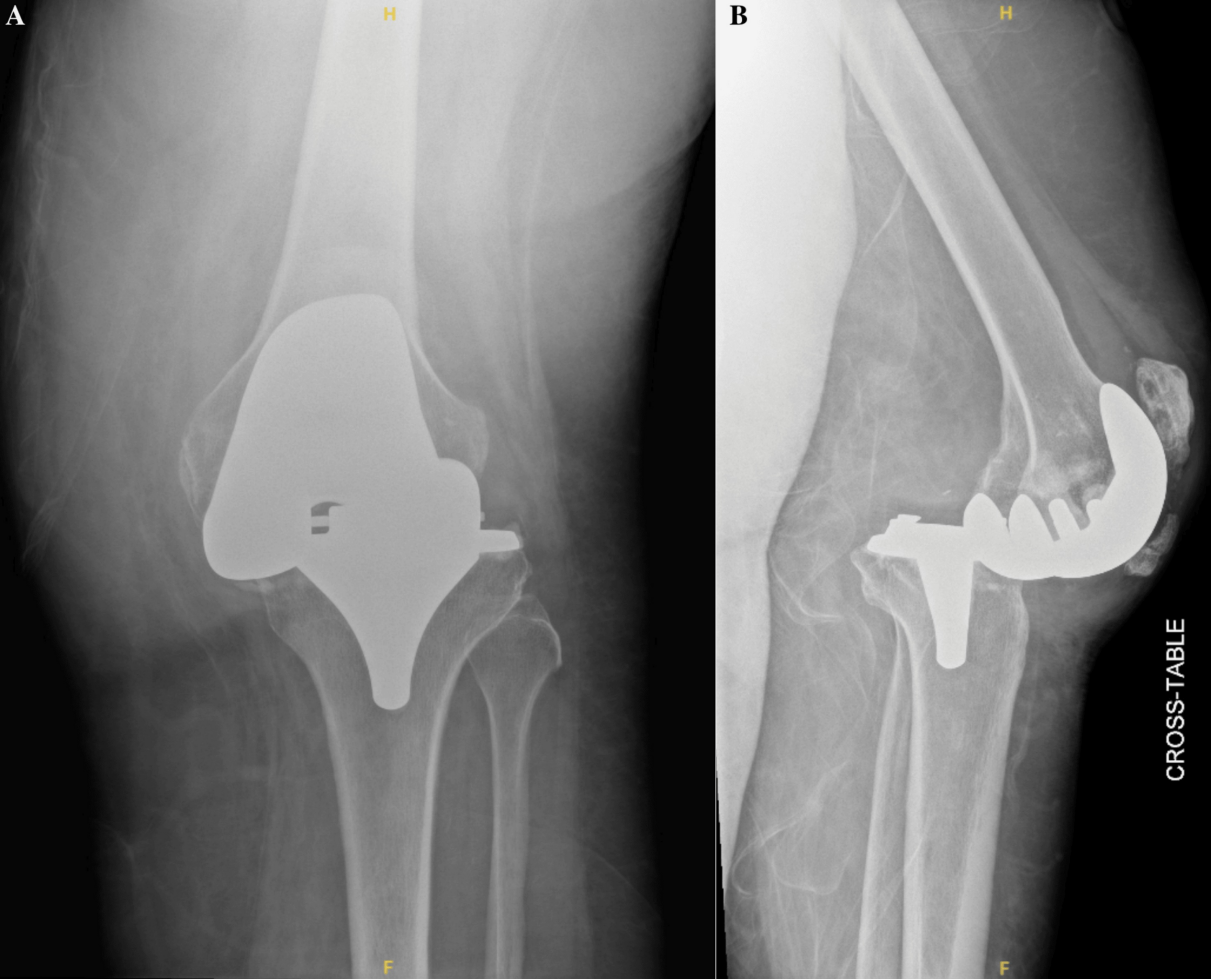
Anteroposterior and cross-table radiographs of the left knee with recurrent periprosthetic knee dislocation. (A) Anteroposterior radiograph of the left knee showing joint malalignment. No evidence of fracture is present. A small joint effusion is present. (B) Cross-table radiograph of the left knee showing a recurrent posterior periprosthetic knee dislocation. Heterotopic ossification is noted on the patellar tendon. A small joint effusion is present.

**Figure 4:**
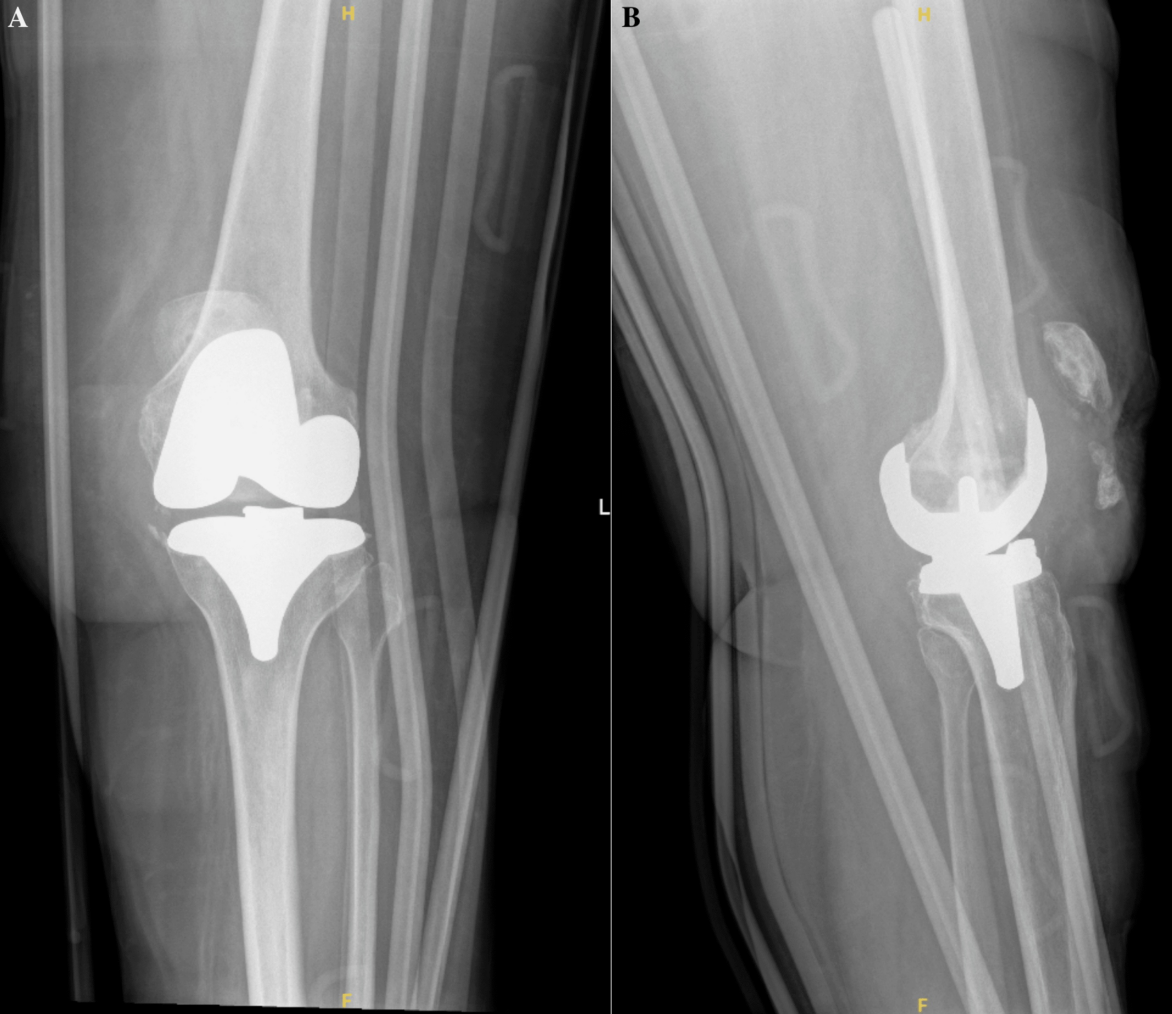
Anteroposterior and cross-table radiographs of the left knee status post-closed reduction under anesthesia. (A) Anteroposterior radiograph of the left knee showing satisfactory reduction and alignment status post-closed reduction under anesthesia. No evidence of fracture can be seen. A small joint effusion is noted. No acute injury is appreciated. An external knee immobilizer is noted. (B) Cross-table radiograph of the left knee showing satisfactory reduction and alignment status post-closed reduction under anesthesia. There is evidence of a small joint effusion, but no acute injury or fracture is noted. Heterotopic ossification is appreciated on the patellar tendon. An external knee immobilizer is noted.

**Figure 5:**
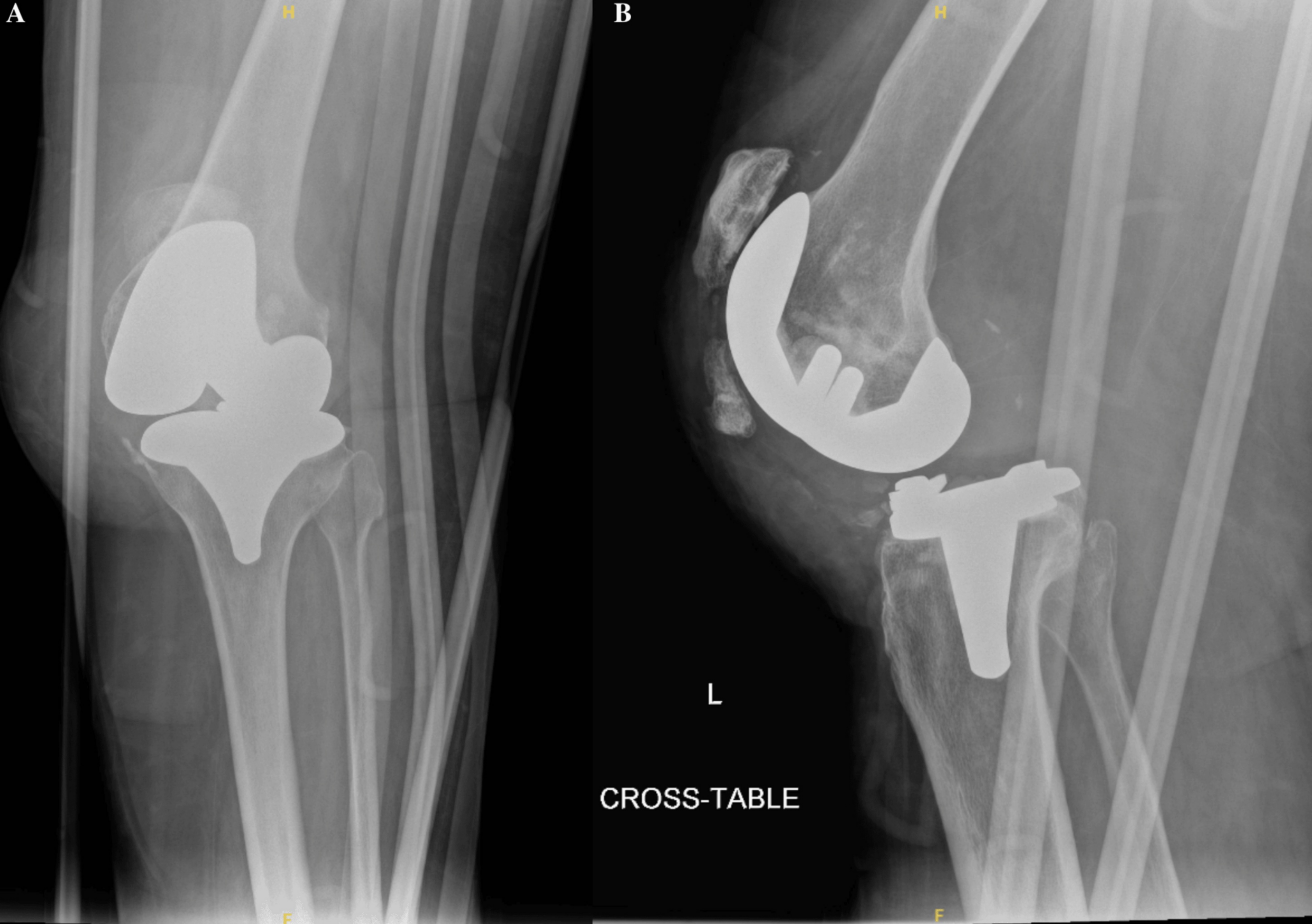
Anteroposterior and cross-table radiograph of the left knee with another recurrent periprosthetic knee dislocation. (A) Anteroposterior radiograph of the left knee showing joint malalignment. A small joint effusion is noted. Moderate soft tissue edema is appreciated. An external knee immobilizer is seen. (B) Cross-table radiograph of the left knee showing recurrent periprosthetic knee dislocation. There is evidence of a small joint effusion. Moderate soft tissue edema is noted. An external knee immobilizer is noted. Heterotopic ossification of the patellar tendon is appreciated.

**Figure 6:**
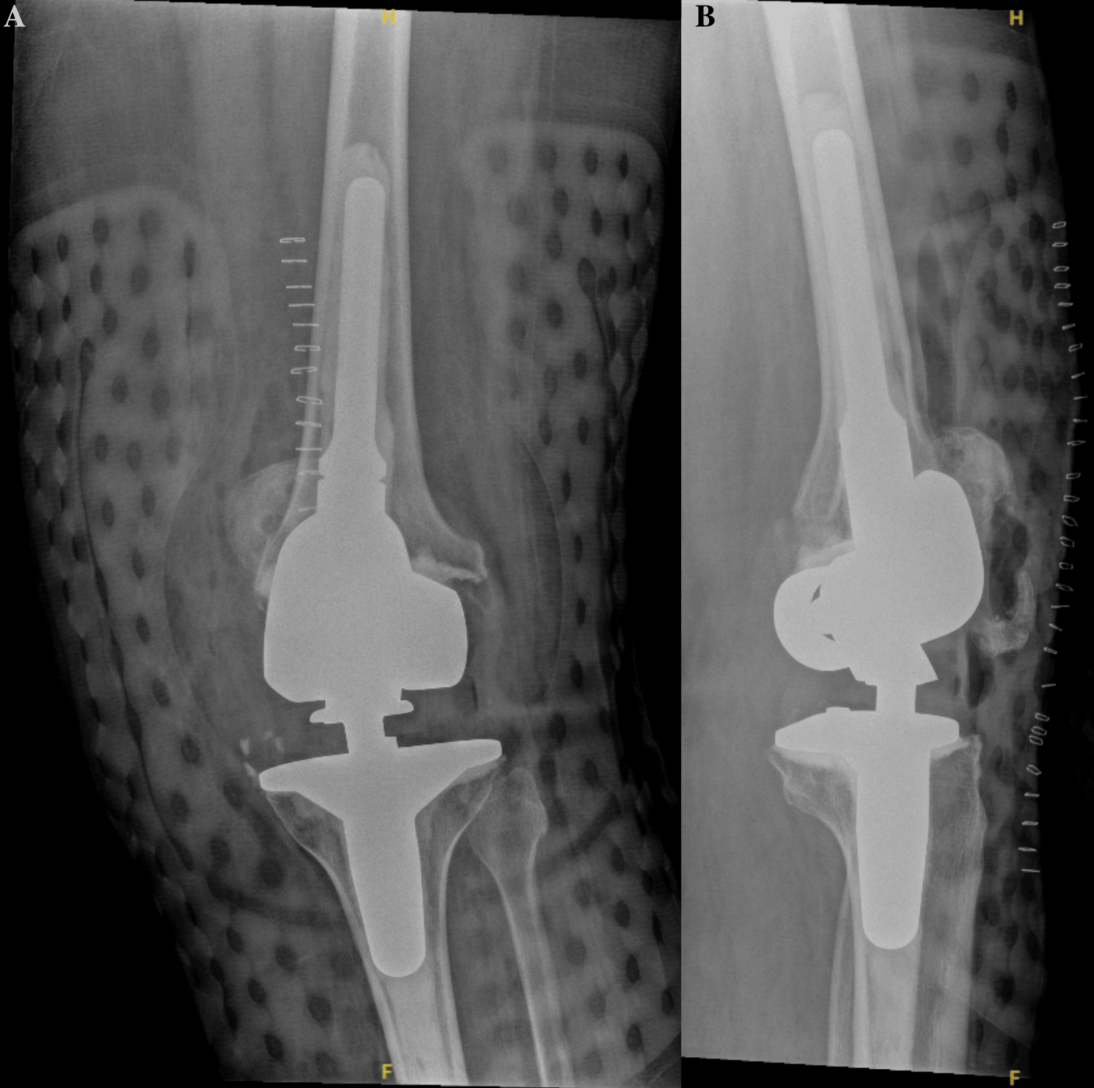
Anteroposterior and cross-table radiographs of the left knee with a cemented revision total knee arthroplasty with a hinged prosthesis. (A) Anteroposterior radiograph of the left knee showing adequate positioning of a cemented revision total knee arthroplasty revision with a hinged prosthesis without evidence of acute complication. Expected post-surgical changes and subcutaneous air are noted. Skin staples are noted. (B) Cross-table radiograph of the left knee showing adequate positioning of a cemented revision total knee arthroplasty revision with a hinged prosthesis without evidence of acute complication. An external post-surgical brace is seen. There are skin staples in place with typical post-surgical changes and subcutaneous air.

A 69-year-old female with an extensive past medical history, including dementia, Ménière’s syndrome, and left TKA, presented to the emergency department (ED) for AMS. During her previous admission for influenza 18 days prior, the patient sustained a fall, resulting in a left PPKD (Figures 1A, 1B). She underwent closed reduction and was placed in a knee immobilizer (Figures 2A, 2B). She was diagnosed with acute metabolic encephalopathy superimposed on dementia, likely related to underlying infectious processes. Upon discharge, she did not have a left PPKD. During her current presentation, her X-rays revealed another left PPKD without evidence of fracture (Figures 3A, 3B). Vascular and neurological structures were unharmed. This subluxation was thought to be due to a fall, so X-rays were obtained from her rehabilitation facility. On these films, the patient was noted to have a left undiagnosed posterior knee subluxation for more than 24 hours. This marked her second left PPKD. The next day, the patient underwent a closed reduction under anesthesia, and she was placed back in her knee immobilizer (Figures 4A, 4B). Again, the patient was found to be neurovascularly intact. During a physical examination, there was a bony abnormality of the left knee with palpation, which resulted in a stat lateral X-ray. The films showed another left PPKD, which marked her third left PPKD in less than one month (Figures 5A, 5B). After evaluating the clinical picture, the operative team decided a revision arthroplasty with a more constrained design would best treat the patient. Four days later, the patient underwent a left cemented revision TKA with a hinged prosthesis (Figures 6A, 6B). The implant was noted to be stable, and the patient had minimal pain postoperatively with no vascular or neurological injury. Due to her ongoing AMS, the patient was not discharged until the fifth postoperative day. Upon outpatient follow-up, the patient reported doing well.

## Discussion

Posterior knee dislocation, known as the subluxation of the proximal tibia posterior to the distal femur, is one of the most common forms of knee subluxation. This type of injury poses a considerable threat to patients as complications of such a dislocation include common peroneal nerve injury (25%), vascular insult (18%), and even amputation (12%) [3,5]. Risk factors that place an individual at increased risk for posterior knee dislocations include obesity, neuropsychiatric disorders, and peripheral neuromuscular diseases [3,6]. In the setting of TKA, risk factors for PPKD include all those stated for knees with native anatomy plus severe (>10°) preoperative valgus/varus deformity and technical errors on the part of the surgical team regarding ligament balance, residual laxity, excessive soft tissue release, and implant malpositioning [6,9-12].

This patient’s dementia and AMS qualified her as a patient with at least one risk factor for dislocation. This increased risk is thought to be because in those with neuropsychiatric and peripheral neuromuscular disorders, desynchronized agonist/antagonist muscle activity places an abnormal strain on the joint [13]. One exemplary mechanism involves unopposed hamstring pull following extensor malfunction or excess lateral patellar maltracking. Although this is a possible occurrence in those with neuropsychiatric or peripheral neuromuscular disorders, it is not entirely exclusive [14]. From the timeline of events for this patient, it is unclear whether the in-patient fall she experienced was a result of or the cause of her original dislocation.

Compared to patients with knees retaining native anatomy, those with TKAs experience knee dislocation considerably less often as the durable plastic and metal are more resistant to damage and the prosthesis itself provides some stability [3,6]. Albeit, it is still possible. Due to newer designs and prostheses, periprosthetic knee dislocations are exceedingly rare (0.15-0.5%), with obesity as the most common comorbidity [3,6]. Even more rare than a periprosthetic knee dislocation is its repetitive recurrence, a complication that has been reported so scarcely in the literature that its rate of occurrence is unknown [15]. For the reported cases, the cause of repeated dislocation was largely attributed to damaged implants, which makes the case presented in this manuscript unique as the patient’s original implant was noted to be stable [16,17].

Although a rare complication, a PPKD poses a serious challenge as manual reduction is often difficult due to muscular forces on the joint. Reduction of such a dislocation can be attempted by imposing traction on the tibia and driving the tibia anteriorly while in a hyperflexed position [6]. This requires patient cooperation and participation, luxuries that are not always afforded by patients experiencing severe pain and possibly less so by those with cognitive impairment or AMS. On top of this, manual reduction also poses further risks to the popliteal artery and the structural ligaments of the knee [6]. For these reasons, operative reduction using the medial parapatellar approach can be used [6]. Regardless of the mode of reduction, repeated failure in terms of joint stability after reduction may require revision TKA, as was seen in this case. For these reasons, posterior knee dislocation in the setting of TKA serves as a rare, however serious, complication that should be considered in the original discussion regarding TKA in those who have risk factors, especially those with neuropsychiatric disorder(s).

Typically, hinged implants are reserved for complex cases of TKA revision due to their highly constrained nature [7]. Despite their high complication rates, these implants are used when large bone defects require reconstruction or when the stability of the adherence of the implant is uncertain [7]. In recent years, the design of these implants has advanced with modularity and rotating bearings, which allows for local joint reconstruction or segmental bone replacement. However, the ceiling for positive outcomes associated with these implants is often masked by the poor general health of the patients receiving them [7]. When choosing an implant for individuals with highly unstable knees, careful selection of prosthesis must be employed. Hinged prostheses are effective options for individuals undergoing revision arthroplasty as they have lower postoperative outcome scores, albeit with similar satisfaction scores. These implants typically do not require reoperation [18]. Given this patient’s complex medical history and subsequent multiple complications involving her original TKA, revision TKA utilizing a hinged prosthesis was determined to be in the patient’s best interest. In this patient, the cost of restraining the joint with a hinged prosthesis was much lower than that of recurrent PPKD due to the aforementioned sequelae.

One point of interest in this case is the recurrence of dislocation despite external knee immobilization following closed reduction. It may seem logical to attribute the dislocation to inadequate bracing, but there is limited literature exploring the role of immobilization following acute knee dislocation/reduction. In fact, Baird et al. reported that there are no available studies comparing knee immobilization for acute knee dislocation versus other treatment options [19]. This area is even more blurry when considering immobilization for an acute dislocation of TKA in a patient with a neuropsychiatric disorder due to the aforementioned possibility of discoordinated agonist/antagonist muscle activity, which may impose abnormal forces on the joint regardless of bracing. Further, it has been reported that complications of immobilization include thigh muscle atrophy, loss of motion, deep vein thrombosis, and delayed return to baseline function, which are all significant consequences in a geriatric patient [19]. Regarding immobilization, more research needs to be done regarding its benefits and complications in acute knee dislocation, subsequent reduction, and rehab. This discussion can also be expanded for its use in periprosthetic dislocations, whether they be isolated, recurrent, and/or in high-risk populations.

Athanasiou et al. and Gomez et al. have separately reported cases describing individual courses of care for a patient with mental impairment suffering from PPKDs requiring revision surgery [4,8]. Outside of these studies, there is scarce literature on the intersection between mental status changes and periprosthetic dislocation, especially those addressed with hinged prostheses. To our knowledge, this is the first report of such an occurrence.

The objective of this manuscript was to address the complications faced by this patient and guide care for physicians who may be challenged by similar cases. With the scarcity of literature regarding multiple topics highlighted within this article, we are hopeful others will continue to add to this topic. Although more research is needed, given this patient’s presumed favorable outcome, the findings presented here may support the use of hinged prostheses in revision TKA under conditions of AMS complicated by complex medical histories.

## Conclusions

Taking into consideration dementia, AMS, and two previously unsuccessful attempts at closed reduction followed by immobilization, a repeat of this standard approach would not be sufficient for the special circumstance of this patient. These comorbidities combined to form a complex problem, which required relatively drastic action to reduce the risk of future devastating consequences and maximize the future quality of life for this patient. This case highlights the limitations surrounding the early identification of PPKD in mentally impaired individuals, the crucial importance of identifying factors that place a patient at increased risk for a PPKD, the adherence to proper operative course for patients with AMS and recurrent PPKD, and the benefit of using hinged prosthesis in complicated cases, such as this one.
